# Cold-resistant lactic acid bacteria in Jerusalem artichoke silage: quality, microbiome and metabolome dynamics during aerobic exposure on the Qinghai-Tibet Plateau

**DOI:** 10.3389/fmicb.2025.1699658

**Published:** 2026-01-21

**Authors:** Xiaoqiang Wei, Lihui Wang, Haiwang Zhang, Long Tan, Shipeng Yang, Jiang Li, Zhenna Liu, Qiwen Zhong, Xuemei Sun

**Affiliations:** 1 Qinghai University, Xining, China; 2 Academy of Agriculture and Forestry Sciences of Qinghai University, Xining, China; 3 Laboratory for Research and Utilization of Germplasm Resources in Qinghai Tibet Plateau, Academy of Agricultural and Forestry Sciences of Qinghai University, Xining, China

**Keywords:** aerobic exposure, cold-resistant lactic acid bacteria, Jerusalem artichoke, microbiome and metabolome, silage quality

## Abstract

Forage scarcity during the cold season poses a major challenge to livestock farming on the Qinghai-Tibet Plateau. Jerusalem artichoke (*Helianthus tuberosus*) offers a promising alternative, but aerobic exposure of its silage leads to nutrient loss and microbial spoilage under low temperatures. This study aimed to evaluate the effects of inoculating cold-resistant lactic acid bacteria (LAB)—*Lactiplantibacillus plantarum* GN02 (homofermentative) and *Levilactobacillus brevis* XN25 (heterofermentative)—on silage quality, microbiome, and metabolome dynamics during aerobic exposure. Silage was prepared from Jerusalem artichoke stems and leaves, treated with sterile water (CK), *Lpl. plantarum* (YZ), *Lv. brevis* (YD), or their mixture (YZD), and ensiled for 60 days at −5 to 8 °C. Samples were analyzed at 0, 7, and 14 days of aerobic exposure (−10 to 5 °C) for fermentation parameters (pH, organic acids, dry matter, water-soluble carbohydrates, crude protein, fibers), microbial communities via 16S rRNA and ITS sequencing, and metabolites using LC–MS-based untargeted metabolomics. Inoculation with *Lpl. plantarum* maintained lower pH (<5), higher lactic acid, dry matter, and water-soluble carbohydrates, while suppressing spoilage bacteria (e.g., *Carnobacterium*, *Citrobacter*) and enriching *Lactobacillus*. Metabolomics revealed upregulated flavonoids and octadecanoids, enhancing antioxidant defenses and downregulating carbohydrate degradation pathways. *Lv. brevis* accelerated spoilage with elevated pH and nutrient loss, whereas the mixture showed intermediate effects. These findings demonstrate *Lpl. plantarum*’s efficacy in mitigating aerobic deterioration, providing a theoretical basis for optimizing silage preservation and supporting sustainable livestock production in high-altitude regions.

## 1 Introduction

The shortage of forage during the cold season is a significant challenge for livestock farming on the Qinghai-Tibet Plateau. Jerusalem artichoke (*Helianthus tuberosus*) is an emerging “food and fodder” economic crop on the Qinghai-Tibet Plateau. Large-scale cultivation can provide an alternative forage source in the cold season. Due to its adaptability to harsh climates, it can grow in barren lands, saline-alkali soils, and gravelly terrains. The above-ground parts of Jerusalem artichoke have high biomass, rich nutritional value, and good palatability. Its stems and leaves show higher crude protein and digestibility than those of potato (*Solanum tuberosum*) and sunflower (*Helianthus annuus*). Consequently, it is recognized as a high-quality roughage and is listed in the China Feed Ingredients Database (Yan et al., 2008; Chen, 2012).

Silage is a feed preservation method that utilizes fermentation by lactic acid bacteria (LAB) (Jin, 2021; Ren et al., 2021). This process generates organic acids to preserve feed freshness and retain nutrients. However, low temperatures on the Qinghai-Tibet Plateau can impair silage quality and slow the fermentation process (Yan et al., 2019). In this study, we previously isolated acid-producing, fast-growing, cold-resistant LAB strains, *Lactiplantibacillus plantarum* GN02 and *Levilactobacillus brevis* XN25, from Jerusalem artichoke silage material on the plateau. These strains, representing homofermentative and heterofermentative LAB, respectively (Wei et al., 2024a), were tested in low-temperature silage of Jerusalem artichoke. We found that *Lpl. plantarum* GN02 increased lactic acid concentration, rapidly lowered pH, enhanced *Lactobacillus* abundance, and inhibited the growth of harmful microorganisms such as *Carnobacterium*, *Desemzia*, and *Enterobacter*, thereby improving the quality of the silage (Wei et al., 2024b). However, during aerobic exposure, silage can deteriorate as harmful microorganisms like yeasts and molds utilize proteins and water-soluble carbohydrates, leading to an increase in temperature, dry matter loss, and a reduction in nutritional value, which affects feed intake and palatability for livestock (Auerbach and Theobald, 2020a). Additionally, certain toxins produced by aerobic microorganisms can enter milk and meat through livestock metabolism, degrading their taste and flavor, and potentially posing health risks to both humans and animals (Ogunade et al., 2018; Gallo et al., 2015). Therefore, controlling the aerobic spoilage of silage is crucial.

The use of LAB has been recognized for enhancing the fermentation quality of silage and mitigating aerobic spoilage (Cai et al., 1999; Filya and Sucu, 2007). Although *Lpl. plantarum* GN02 has demonstrated high efficiency in lactic acid production (Wei et al., 2024b), studies indicated that it might negatively affect aerobic stability (Andrae et al., 2001; Cole et al., 2007). Researchers were currently investigating various heterofermentative LAB strains, including *Lv. brevis* (Drouin et al., 2019), *Lentilactobacillus buchneri* (Contreras-Govea and Muck, 2006; Bai et al., 2020), *Lentilactobacillus hilgardii* (Duniere et al., 2017), *Lentilactobacillus kefiri* (Edgar, 2010), and *Lacticaseibacillus rhamnosus* (Ferraretto and Shaver, 2012), to address aerobic spoilage in silage. Notably, *Lv. brevis* has shown potential as a silage inoculant by enhancing the aerobic stability and acetic acid concentration of paper mulberry silage while consistently inhibiting the growth of molds and yeasts (Wiedmann, 2003). However, under anaerobic storage conditions, the nutrient preservation and acidification levels achieved by heterofermentative pathways are generally lower than those of homofermentative pathways (Wilkinson and Davies, 2013; Guan et al., 2021). Balancing the roles of these LAB types in silage remains a critical focus in the development of effective silage inoculants.

The metabolic changes during aerobic exposure of silage are complex and dynamic, involving the synthesis, degradation, and transformation of various organic compounds. Under aerobic conditions, carbohydrates and proteins are utilized by aerobic microorganisms, producing toxic metabolites. This process may also trigger the synthesis of other antioxidant substances or enhance the expression of antioxidant enzymes (Corsetti et al., 2004). Analyzing the metabolites during aerobic exposure can improve our understanding of the silage bioprocess (Guan et al., 2020) and provide insights into the mechanisms of protein hydrolysis during exposure. Metabolomic studies have shown that LAB in forage silage can produce additional organic acids, such as p-coumaric acid, hydroxycinnamic acid, p-hydroxybenzoic acid, 3-hydroxydecanoic acid, and 3-phenyllactic acid (Broberg et al., 2007). Beyond organic acids, some metabolites have antimicrobial properties, including acetaldehyde, ethanol, hydrogen peroxide, 2,3-butanedione, and exopolysaccharides (Driehuis and Elferink, 2000; Sjögren et al., 2003). Metabolomics can detect and quantify many other compounds in silage. In a study by Hu et al. (2020), 196 metabolites, mainly organic acids, polyols, ketones, and aldehydes, were detected in silage samples exposed to aerobic conditions for 3 days after LAB inoculation. These metabolites, which have antimicrobial properties, were predominant when LAB and enterococci were dominant in the silage. Currently, there is limited research on the metabolomics of aerobic exposure in silage, highlighting the need for in-depth exploration of the dynamic changes in metabolites and the interactions between microorganisms and metabolites during this period.

This study focuses on Jerusalem artichoke silage inoculated with two cold-resistant LAB strains and stored for 60 days under natural conditions. After aerobic exposure, we utilized high-throughput microbial sequencing and liquid chromatography-mass spectrometry (LC–MS) untargeted metabolomics to investigate the changes in microorganisms and metabolites during the aerobic exposure phase of low-temperature silage. We also assessed the quality changes in the silage. The goal was to clarify the extent to which the inoculation of cold-resistant LAB mitigates aerobic spoilage under low-temperature conditions on the Qinghai-Tibet Plateau. Additionally, we aimed to explore the interactions between silage quality changes and microbial metabolite succession. The findings of this study provide a theoretical foundation for silage practices on the Qinghai-Tibet Plateau and offer important guidance for the sustainable and stable development of livestock farming in the region.

## 2 Materials and methods

### 2.1 Silage preparation

The experimental material used was the Jerusalem artichoke variety “Qingyu No. 2,” which was independently bred by the Academy of Agriculture and Forestry Sciences of Qinghai University. The planting took place on April 20, 2024, in the experimental field of the Horticultural Innovation Base at Academy of Agriculture and Forestry Sciences of Qinghai University, Xining, Qinghai Province (E101°44′57.7572″, N36°43′8.346″, altitude 2266.6 m). On October 21, 2024, the above-ground stems and leaves were harvested and chopped into pieces approximately 2 ± 1 cm in length for silage. The cold-resistant LAB used in the experiment were *Lpl. plantarum* GN02 and *Lv. brevis* XN25, previously screened in the laboratory, with NCBI accession numbers OP740787 and OP740791, respectively. In our preliminary work, we evaluated the low-temperature traits of *Lactiplantibacillus plantarum* GN02 and *Levilactobacillus brevis* XN25 by measuring their growth curves and acid-production capacity; the results showed that at 4 °C both strains exhibited relatively high growth rates and robust acid production (Wei et al., 2024a,b). Before the experiment, the LAB strains stored at −80 °C were thawed at 4 °C. Cultures were sub-cultured twice in MRS broth and incubated without shaking for 18 h. The viable cell count was measured using OD values, and the cultures were centrifuged (4,000 rpm, 5 min). The bacterial cells were resuspended in sterile water to a final concentration of approximately 1.0 × 10^8^ cfu/mL. Five hundred grams of the chopped Jerusalem artichoke material were randomly sampled, and the prepared LAB inoculant was uniformly sprayed onto the material using a sterile sprayer (inoculation rate of 1.0 × 10^6^ cfu/g fresh matter). The treated material was packed in vacuum-sealed polyethylene bags (280 mm × 350 mm, Zhejiang, China) using a vacuum sealer (14891, Deli, Shandong, China). Bags were stored under local conditions (ensiling −5 to 8 °C; exposure −10 to 5 °C; 40–65% RH) for 60 days before aerobic exposure. During exposure, bag openings were wrapped with double-layer medical gauze to prevent contamination and thereby reduce experimental error. Four treatments were established in the experiment: YS (5 mL sterile water), YD (*Lv. brevis* XN25, 1.0 × 10^6^ cfu/g FM), YZ (*Lpl. plantarum* GN02, 1.0 × 10^6^ cfu/g FM), and YZD (*Lv. brevis* XN25 + *Lpl. plantarum* GN02 mixture, 1.0 × 10^6^ cfu/g FM). Each treatment was replicated three times, and samples were taken at 0, 7, and 14 days of aerobic exposure (0 days being the 60-day fermented silage material), resulting in 36 bags in total (3 exposure days × 4 treatments × 3 replicates). The samples were analyzed for silage quality indicators and microbial communities. Additionally, samples exposed to air for 14 days were used to detect the types and quantities of metabolites.

### 2.2 Determination of silage quality indicators

Twenty gram of the sample was homogenized with 180 mL of distilled water using a blender at high speed for 30 s. The mixture was then filtered through four layers of medical gauze to collect the filtrate. The filtrate was centrifuged (5,000 rpm, 10 min), and the pH was measured using a glass electrode pH meter (pH S-2F, LEICI, Shanghai, China). The filtrate was stored at −20 °C for subsequent analysis of lactic acid (LA), acetic acid (AA), propionic acid (PA), and ammonia nitrogen (NH_3_-N) concentrations. The concentrations of LA, AA, and PA were determined using high-performance liquid chromatography (HPLC) (Li et al., 2018). The NH_3_-N concentration was measured using a Plant Ammonia Nitrogen Content Assay Kit, following the manufacturer’s instructions (Beijing Boxbio Science & Technology Co., Ltd.), with absorbance read at 570 nm using a microplate reader (EPOCH2, BioTek, Vermont, United States). The primary output of the kit is given on a fresh-matter basis. In the present study, NH_3_-N values were first obtained in the original unit provided by the kit and then converted to a DM basis (g/kg DM) according to the actual DM content of each silage sample (Kozloski et al., 2006). Total nitrogen (TN, g/kg DM) was calculated from crude protein (CP) as TN = CP/6.25 (Baur and Ensminger, 1977). The proportion of ammonia nitrogen in total nitrogen (NH_3_-N/TN, %) was used as an indicator of protein degradation during aerobic exposure (Li et al., 2024). The DM-based NH_3_-N values, together with TN and NH_3_-N/TN (%) for each treatment and sampling day, are presented in Supplementary Table S1. An additional 300 g sample was dried in an oven at 65 °C for 48 h to a constant weight to determine the dry matter (DM) content using an electronic balance (PTX-FA210S, HuaZhi, Connecticut, United States). The dried sample was ground and passed through a 1 mm sieve for subsequent analyses of CP, water-soluble carbohydrates (WSC), neutral detergent fiber (NDF), and acid detergent fiber (ADF) contents. CP concentration was measured using the Kjeldahl method, following the Association of Official Analytical Chemists (Baur and Ensminger, 1977) guidelines, with a Kjeldahl nitrogen analyzer (K9860, Hainon, Shandong, China). WSC concentration was analyzed using the anthrone reagent method (Thomas, 1977). The NDF and ADF contents were determined according to the method of Van Soest et al. (1991), using a fiber analyzer (F800, Hainon, Shandong, China).

### 2.3 Analysis of microbial communities

After collection, silage samples were flash-frozen in liquid nitrogen and stored at −80 °C. Bacterial and fungal DNA were extracted from the silage using the MagPure Soil DNA LQ Kit (Yesen, Shanghai, China) according to the manufacturer’s instructions. DNA concentration was measured using a spectrophotometer (NanoDrop 2000), and the integrity was assessed by agarose gel electrophoresis. In 25 μL reactions, universal primers amplified the bacterial 16S rRNA V3–V4 region and the fungal ITS1 region. The bacterial primers used were 343F (5′-TACGGRAGGCAGCAG-3′) and 798R (5′-AGGGTATCTAATCCT-3′). The fungal primers used were ITS1F (5′-CTTGGTCATTTAGAGGAAGTAA-3′) and ITS2 (5′-GCTGCGTTCTTCATCGATGC-3′). Specific barcodes were incorporated into the reverse primers, and Illumina sequencing adapters were attached to both primers.

The quality of the amplicons was assessed using gel electrophoresis. PCR products were purified with Agencourt AMPure XP beads and quantified using the Qubit dsDNA Assay Kit. After adjusting the concentrations, sequencing was performed on the Illumina NovaSeq6000 platform using paired-end reads of 250 bases each. The raw sequencing data were presented in FASTQ format. Paired-end reads were then preprocessed using cutadapt software to identify and remove adapters. After trimming, the reads were filtered to remove low-quality sequences, denoised, and merged using DADA2 (Callahan et al., 2016), with default parameters in QIIME2, to detect and remove chimeras. The software generated representative reads and an abundance table for the Amplicon Sequence Variants (ASVs). Representative reads for each ASV were selected using the QIIME 2 package. These representative reads were annotated and compared against the Silva database version 138 (16S rDNA) and Unite (ITS) using the q2-feature-classifier with default parameters. Microbial diversity was assessed using α-diversity indices, including Chao1 richness and the Shannon diversity index. A Unifrac distance matrix, calculated using QIIME software, was used for unweighted Unifrac Principal Coordinates Analysis (PCoA). Microbial biomarkers were identified using Linear Discriminant Analysis (LDA) with an LDA score threshold of 4 (Weiss, 2017).

In this study, we employed Illumina MiSeq next-generation sequencing (NGS) for 16S rRNA and ITS amplicon sequencing. Although third-generation sequencing platforms, such as PacBio and Oxford Nanopore, offer advantages including long read lengths, reduced GC bias, and single-molecule sequencing, NGS was chosen due to its high throughput, relatively low cost, and well-established pipelines for short amplicon regions. We acknowledge that NGS has certain limitations, such as shorter read lengths, potential GC bias, and limited resolution for distinguishing highly similar sequences. However, these limitations are unlikely to affect the main conclusions of this study, as our analysis focuses on dominant and common microbial taxa, which are reliably captured by MiSeq short-read sequencing.

### 2.4 Metabolite analysis

The reagents used for metabolite analysis were all of HPLC grade. Water, methanol, acetonitrile, and formic acid were purchased from Thermo Fisher Scientific (United States), and L-2-chlorophenylalanine was obtained from Shanghai Hengchuang Biotechnology Co., Ltd. Prior to use, reagents were pre-cooled to −20 °C. Samples stored at −80 °C were thawed at room temperature. Weighed 60 mg of sample was placed in a 1.5 mL Eppendorf tube with two small steel balls. Each sample was mixed with 600 μL of methanol–water solution (7:3, V/V), containing L-2-chlorophenylalanine (4 μg/mL). The mixture was pre-cooled at −40 °C for 2 min and then ground using a grinder (60 Hz, 2 min). The resulting mixture was sonicated in an ice-water bath for 30 min and left to stand at −40 °C overnight. On the second day, the samples were removed from −40 °C, centrifuged at 12000 rpm for 10 min at 4 °C. The supernatant (150 μL) was filtered through a 0.22 μm filter into an LC vial and stored at −80 °C until LC–MS analysis. QC samples were prepared by mixing equal volumes of the extracts from all samples.

Metabolomic data analysis was conducted by Shanghai Luming Biotechnology Co., Ltd. (Shanghai, China). The analysis utilized a Q-Exactive mass spectrometer equipped with a heated electrospray ionization (ESI) source (Thermo Fisher Scientific, Waltham, MA, United States) connected to an ACQUITY UPLC I-Class plus system (Waters Corporation, Milford, MA, United States). Both ESI positive and negative ion modes were employed. Separation was achieved using an ACQUITY UPLC HSS T3 column (1.8 μm, 2.1 × 100 mm) in both positive and negative ion modes. A binary gradient elution system was employed with solvent A (water with 0.1% formic acid, v/v) and solvent B (acetonitrile with 0.1% formic acid, v/v) using the following gradient: 0.01 min, 5% B; 2 min, 5% B; 4 min, 30% B; 8 min, 50% B; 10 min, 80% B; 14 min, 100% B; 15 min, 100% B; 15.1 min, 5% B; 16 min, 5% B. The flow rate was 0.35 mL/min with a column temperature of 45 °C. All samples were maintained at 10 °C during analysis. Injection volume was 2 μL. Mass spectrometry covered a mass range of 100–1,000 m/z with MS scan resolution set at 70,000 and HCD MS/MS scan resolution at 17,500. Collision energies were set at 10, 20, and 40 eV. Operating parameters for the mass spectrometer were as follows: spray voltage 3,800 V (+) and 3,200 V (−); sheath gas flow rate 35 arbitrary units; auxiliary gas flow rate 8 arbitrary units; capillary temperature 320 °C; auxiliary gas heater temperature 350 °C; SS-lens RF level 50.

The raw LC–MS data were processed using Progenesis QI V2.3 software (Nonlinear Dynamics, Newcastle, UK). This included baseline filtering, peak identification, integration, retention time correction, peak alignment, and normalization. A precursor tolerance of 5 ppm, a product tolerance of 10 ppm, and a product ion threshold of 5% were primarily used. Compound identification was based on accurate mass-to-charge ratio (m/z), secondary fragmentation, and isotopic distribution. Databases used included the Human Metabolome Database (HMDB), Lipidmaps (V2.3), Metlin, and an in-house database. The extracted data were further processed by removing peaks with missing values (ion intensity = 0) in more than 50% of the groups. Zero values were replaced with half of the minimum value, and compounds were filtered based on qualitative results. Compounds with a score below 36 (out of 60) were considered inaccurate and were removed. A data matrix was constructed by combining positive and negative ion data.

The matrix was imported into R software for principal component analysis (PCA) to observe the overall distribution of samples and the stability of the analysis process. Orthogonal partial least squares discriminant analysis (OPLS-DA) and partial least squares discriminant analysis (PLS-DA) were used to differentiate metabolites between different groups. To prevent overfitting, the model quality was assessed using 7-fold cross-validation and 200 permutations of response testing (RPT). The variable importance in projection (VIP) values obtained from the OPLS-DA model were used to rank the overall contribution of each variable to group discrimination. A two-tailed *T*-test was further used to determine if there were significant differences in metabolites between the groups.

### 2.5 Statistical analysis

Basic statistical analysis of quality data was performed using Excel 2019. IBM SPSS 27.0 software was used for statistical analysis of the quality parameters of aerobic exposure in silage, with results presented as means and standard errors. One-way ANOVA and Duncan’s multiple range test were applied, with *p* < 0.05 indicating significance. Bar charts of the results were generated using Origin 2022 software. Microbial and metabolite sequencing data were analyzed and visualized using the Oebiotech cloud platform.^1^ In the analysis of microbial and metabolite correlations, bacterial ASV data from 14 days of aerobic exposure were used.

## 3 Results

### 3.1 Silage quality analysis

**Figure 1:**
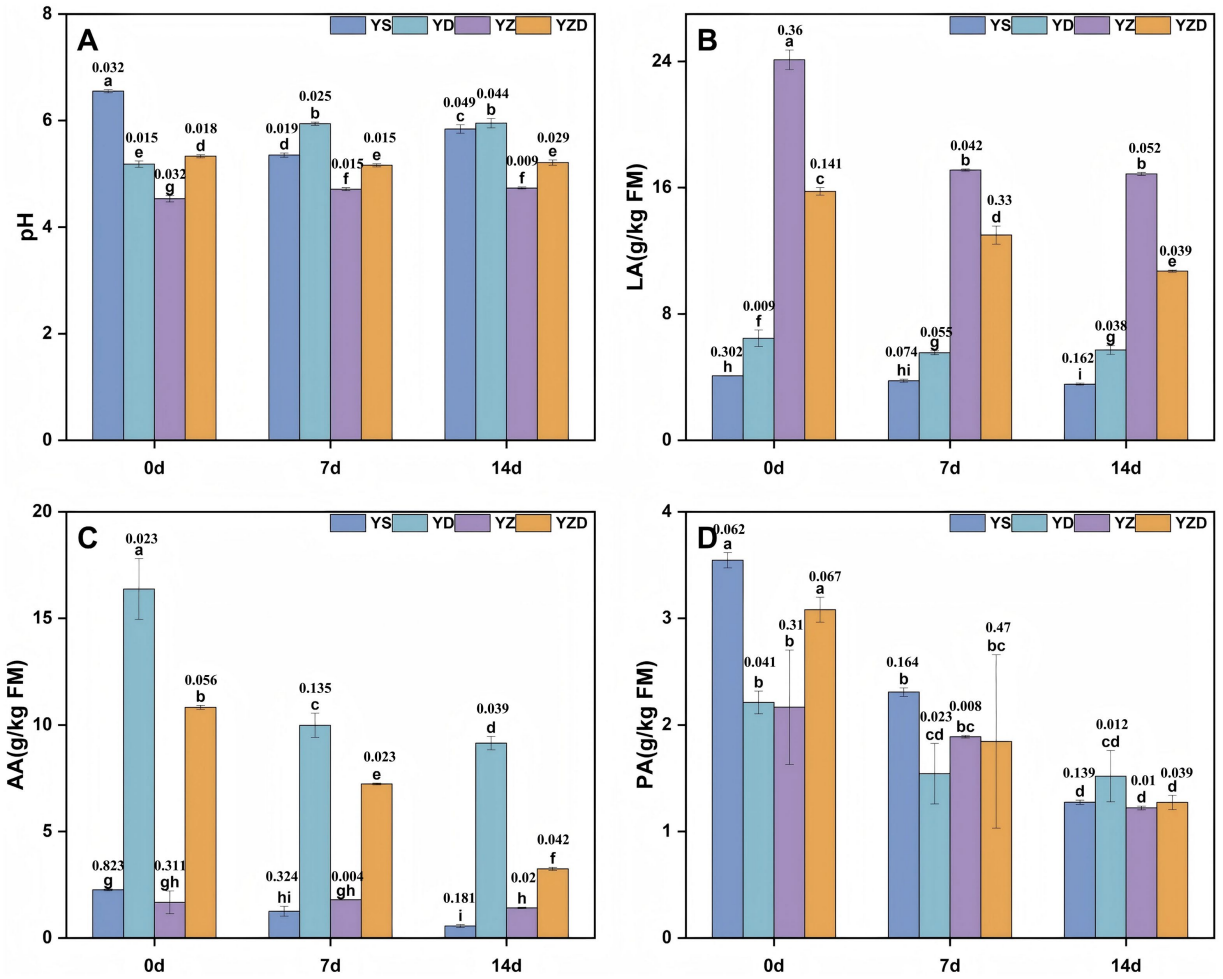
Changes in pH **(A)**, LA **(B)**, AA **(C)**, and PA **(D)** during aerobic exposure of silage. pH, Potential of hydrogen; LA, Lactic acid; AA, Acetic acid; PA, Propionic acid; YS, sterile water; YD, *L. brevis*; YZ, *L. plantarum*; YZD, *L. brevis* + *L. plantarum*. Numbers in the figure denote SEM (*n* = 3). Different lowercase letters denote significant differences between treatments on the same or different aerobic exposure days (*p* < 0.05), while identical lowercase letters indicate no significant difference (*p* > 0.05). Same below.

During aerobic exposure, the pH levels of all groups changed (Figure 1A). The pH in the YS group first decreased and then increased, while in the YD and YZ groups it increased, and in the YZD group it decreased. At 7 and 14 d, within-group differences were not significant (except YS), whereas between-group differences were significant. The YZ group had a significantly lower pH (4.71, 4.73) compared to the other groups, while the YD group had a significantly higher pH (5.94, 5.95). The LA concentration in all groups gradually decreased over time (Figure 1B). Similar to the pH results, there were significant differences in LA concentration between groups at 7 and 14 days, with the YZ group showing significantly higher LA concentration (17.11 g/kg, 16.87 g/kg FM) than the others. The AA concentration also continuously declined over time in all groups (Figure 1C), with significant differences between groups at 7 and 14 days. The YD group had a significantly higher AA concentration (9.98 g/kg, 9.14 g/kg FM) compared to the other groups. Notably, the PA concentration in all groups decreased gradually over time (Figure 1D), and by 14 days, there were no significant differences between groups, although the YD group had slightly higher values.

**Figure 2:**
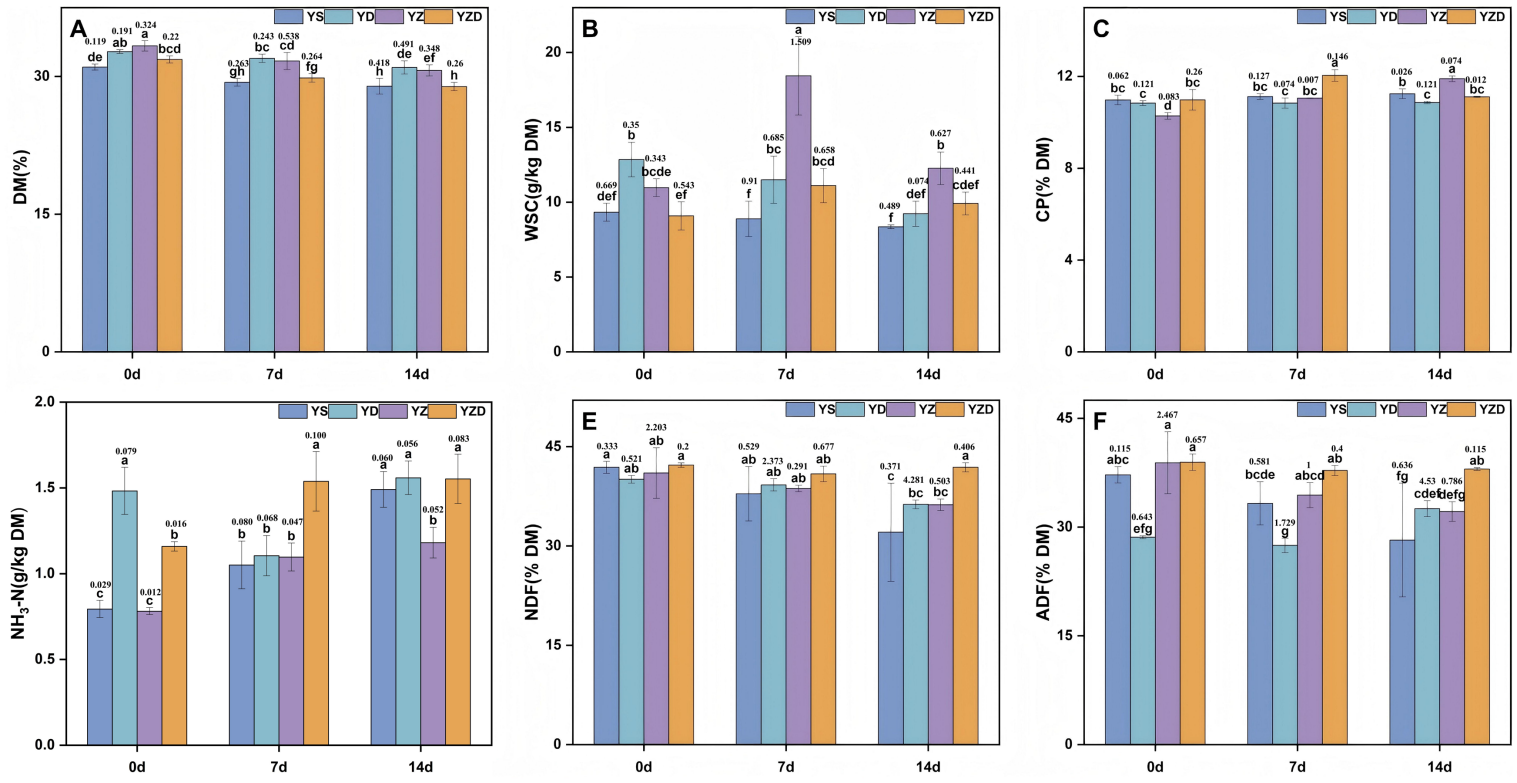
Changes in DM **(A)**, WSC **(B)**, CP **(C)**, NH_3_-N **(D)**, NDF **(E)**, and ADF **(F)** during aerobic exposure of silage. DM, dry matter; WSC, water-soluble carbohydrates; CP, crude protein; NH_3_-N, ammonia nitrogen; NDF, neutral detergent fiber; ADF, acid detergent fiber.

The DM content in all groups gradually decreased over time (Figure 2A). At 7 and 14 days, the YD and YZ groups had significantly higher DM content compared to the YS and YZD groups. The WSC concentration varied differently over time among the groups (Figure 2B); it decreased monotonically in YS and YD, but showed a rise-then-fall pattern in YZ and YZD. At 7 and 14 days, the YZ group had significantly higher WSC concentration (18.44 g/kg, 12.26 g/kg DM) than the other groups, while there was no significant difference between the YD and YZD groups, which were higher than the YS group. The CP concentration remained relatively unchanged over time in the YS and YD groups (Figure 2C), increased in the YZ group, and first increased then decreased in the YZD group. At 7 days, the YZD group had significantly higher CP concentration (12.04% DM) compared to the other groups, while at 14 days, the YZ group had significantly higher CP concentration (11.89% DM). The NH_3_-N concentration gradually increased over time in the YS and YZ groups, decreased and then increased in the YD group, and increased and then decreased in the YZD group (Figure 2D). At 7 days, the YZD group showed the highest NH₃-N concentration (1.54 g/kg DM), which was significantly greater than the other treatments. By 14 days, the YS, YD, and YZD groups had similarly elevated NH_3_-N concentrations (1.49–1.56 g/kg DM), all significantly higher than YZ (1.18 g/kg DM). Notably, there was no significant difference in NH_3_-N concentration between 7 and 14 days in the YZ group. Across treatments, TN remained relatively stable during aerobic exposure (16.44–19.26 g/kg DM), whereas NH_3_-N/TN ranged from 4.53 to 8.97% and thus remained below the 10% threshold that is generally considered indicative of good-quality silage (Supplementary Table S1). Except for the YS group, the NDF content showed little variation over time in all groups (Figure 2E). At 7 days, there were no significant differences between groups, but at 14 days, the YZD group had significantly higher NDF content compared to the others. The ADF content decreased over time in the YS and YZ groups (Figure 2F), increased in the YD group starting at 7 days, and showed no significant change in the YZD group. At 7 days, the YD group had significantly lower ADF content than the other groups, while at 14 days, the YZD group had significantly higher content.

### 3.2 Analysis of microbial community diversity in silage

**Figure 3:**
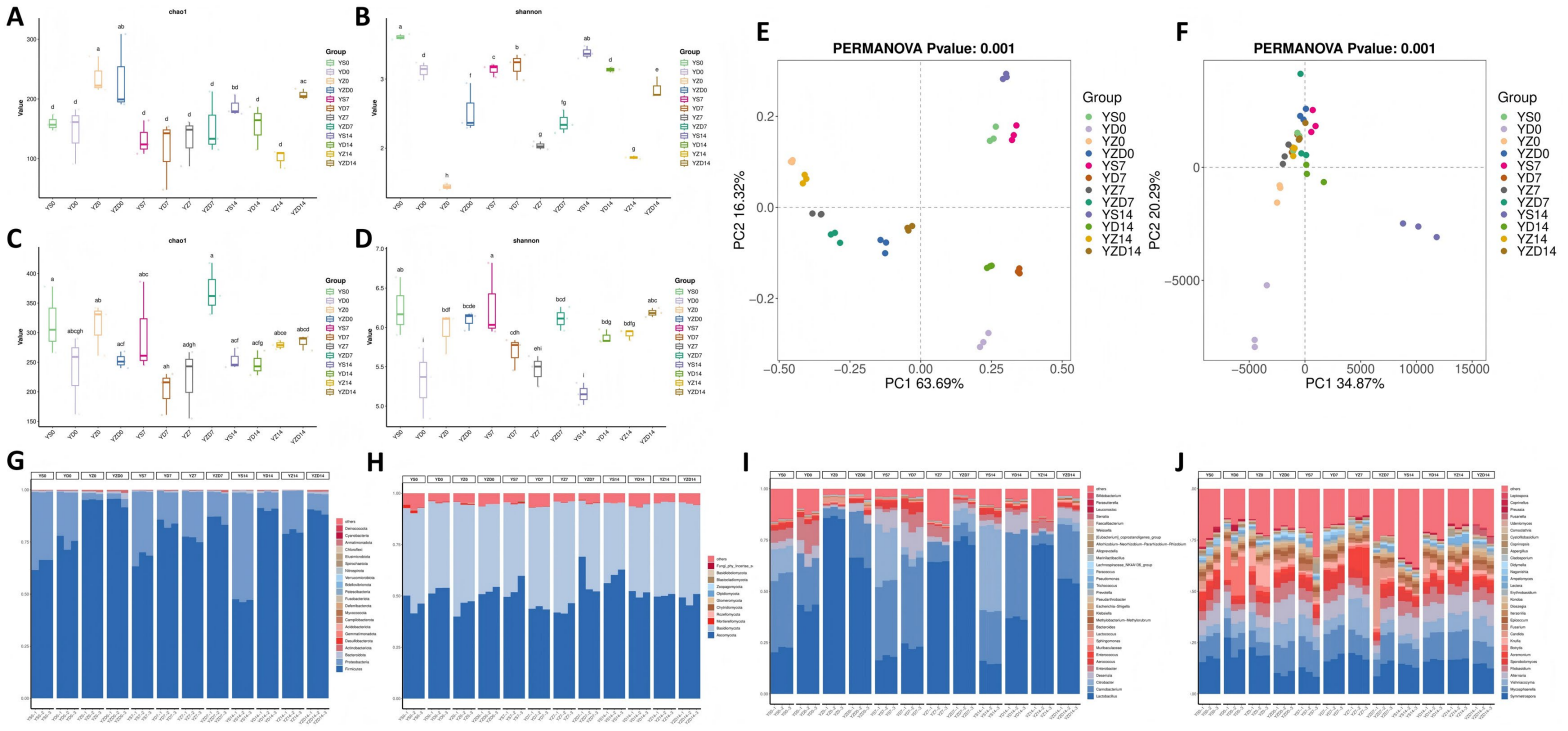
Microbial diversity analysis during aerobic exposure of silage. Alpha diversity analysis for bacteria **(A,B)** and fungi **(C,D)**; principal coordinate analysis (PCoA) of bacterial (**E**, Euclidean method) and fungal (**F**, Bray-Curtis method) community structures, ANOSIM test (*p* < 0.05); relative abundance of bacteria **(G,I)** and fungi **(H,J)** at the phylum and genus levels, the numbers 0, 7, and 14 represent aerobic exposure days.

The α-diversity indices, Chao1 and Shannon, were used to describe the richness and diversity of bacterial and fungal communities. The Chao1 index estimates the number of ASVs in a community, while the Shannon index measures diversity within the community. Higher values of both indices indicate greater richness and diversity. For bacteria (Figures 3A,B), the Chao1 index showed no significant changes over time in the YS and YD groups, decreased gradually in the YZ group, and initially decreased then increased in the YZD group. At 7 days of aerobic exposure, there were no significant differences in the Chao1 index among the groups; however, by 14 days, the YZD group had significantly higher values than the other groups, while the YZ group had the lowest, indicating the lowest community richness in the YZ group during aerobic exposure. The Shannon index varied significantly over time, with the YS group initially decreasing then increasing, the YD group initially increasing then decreasing, and both the YZ and YZD groups showing increasing trends. At 7 and 14 days, the Shannon index in the YZ group was significantly lower than in the other groups, indicating the lowest community diversity in the YZ group during aerobic exposure. For fungi (Figures 3C,D), there were no significant differences in the Chao1 index among the groups during aerobic exposure, suggesting similar fungal community richness. The Shannon index decreased in the YS group from day 7, showed an increasing trend in the YD group, first decreased then increased in the YZ group, and showed no significant change in the YZD group. At 7 days, the Shannon index was lower in the YD and YZ groups compared to the other two groups. Notably, at 14 days, there were no significant differences in the Shannon index among the YD, YZ, and YZD groups, which were all significantly higher than the YS group, indicating that fungal community diversity tended to stabilize in the later stages of aerobic exposure.

Principal Coordinate Analysis (PCoA) was performed to analyze the microbial communities in Jerusalem artichoke silage during aerobic exposure. In bacteria (Figure 3E), the groups were widely dispersed, indicating significant differences between them. The YS and YD groups separated along the PC2 axis, while the YZ and YZD groups separated along the PC1 axis. This suggests that the type of bacterial inoculant and exposure time were major factors contributing to these differences. Notably, the YZ and YZD groups were more clustered compared to the YS and YD groups. For fungi (Figure 3F), the separation between groups was less distinct, with only the 0-day YD group and the 14-day YS group showing a notable distance apart. An ANOSIM test based on Euclidean distance indicated that the differences in bacterial and fungal community structures between groups were significantly greater than within groups (*R*^2^ = 0.98, *p* = 0.001; *R*^2^ = 0.99, *p* = 0.001), confirming the reliability of the PCoA clustering. These findings highlight that the type of inoculant and the duration of aerobic exposure significantly influence microbial community changes in Jerusalem artichoke silage.

### 3.3 Silage microbial species composition

Using the Illumina MiSeq sequencing platform, 16S rRNA sequencing was performed on 36 samples. For bacteria, the raw reads ranged from 78,026 to 81,712, clean tags ranged from 58,667 to 76,196, and valid tags ranged from 42,384 to 66,723. The number of ASVs ranged from 48 to 308. Based on 100% sequence similarity, the sequences were classified into 22 phyla, 45 classes, 114 orders, 203 families, 383 genera, and 630 species. For fungi, the raw reads ranged from 78,145 to 81,887, clean tags ranged from 66,115 to 78,064, and valid tags ranged from 66,115 to 78,064. The number of ASVs ranged from 155 to 418. Classification revealed 13 phyla, 35 classes, 84 orders, 184 families, 392 genera, and 615 species.

At the phylum level (Figure 3G), the dominant bacterial phyla across all groups at each time point were Firmicutes and Proteobacteria. On day 0, Firmicutes was the dominant phylum in all groups, with relative abundances of 64.71, 74.91, 95.22, and 94.38%, respectively. As aerobic exposure progressed, by day 7, the YS group’s Firmicutes and Proteobacteria abundances remained relatively stable. In the YD group, Firmicutes increased slightly to 83.71%, while Proteobacteria decreased to 15.25%. In the YZ and YZD groups, Firmicutes abundance decreased to 76.54 and 85.83%, respectively, while Proteobacteria began to occupy a notable portion of the community, reaching 22.61 and 12.93%. By day 14, the YS group exhibited a balance between Firmicutes and Proteobacteria, with relative abundances of 46.93 and 51.62%, respectively, with Proteobacteria slightly more abundant. In the YD group, Firmicutes further increased to 90.70%. In the YZ and YZD groups, the changes in Firmicutes were minimal compared to day 7, with a decrease in Proteobacteria abundance in the YZD group to 8.46%. At the fungal phylum level (Figure 3H), the dominant phyla across all groups at each time point were Ascomycota and Basidiomycota, which together comprised over 90% of the total community. As aerobic exposure continued, the relative abundances of these two phyla began to shift, though the changes were less pronounced compared to bacteria. On day 0, the YS group had nearly equal abundances of Ascomycota and Basidiomycota (45.99 and 46.01%). In the YD and YZD groups, Ascomycota was more abundant than Basidiomycota, while in the YZ group, Ascomycota was less abundant than Basidiomycota. By day 7, Ascomycota increased and Basidiomycota decreased in the YS and YZD groups, with opposite trends observed in the YD and YZ groups. Ascomycota was more abundant in the YS and YZD groups compared to the YD and YZ groups, where Basidiomycota was more prevalent. By day 14, Ascomycota continued to increase in the YS group (59.79%), while Basidiomycota continued to decrease (35.50%). The YD, YZ, and YZD groups showed trends opposite to those observed on day 7.

At the genus level, the microbial community compositions differed among the groups. For bacteria (Figure 3I), on day 0, the dominant genera in the YS and YD groups were *Carnobacterium* (35.78, 23.17%), *Lactobacillus* (21.90, 42.40%), *Enterobacter* (5.61, 12.65%), and *Desemzia* (3.41, 6.24%), with *Citrobacter* also being prominent in the YS group (13.52%). In the YZ and YZD groups, *Lactobacillus* was the dominant genus (85.94, 64.84%), with *Carnobacterium* (22.26%) and *Desemzia* (3.89%) also being significant in the YZD group. By day 7, in the YS group, the abundances of *Lactobacillus* (17.81%) and *Carnobacterium* (36.49%) remained stable, while *Citrobacter* (26.47%) and *Desemzia* (8.65%) increased, and *Enterobacter* (1.41%) decreased. In the YD group, *Lactobacillus* (23.16%) decreased significantly, while *Carnobacterium* (42.27%) and *Desemzia* (12.25%) increased, and *Enterobacter* (7.55%) decreased. In the YZ and YZD groups, *Lactobacillus* remained dominant (64.42, 74.63%) but decreased in abundance. The YZD group had higher levels than the YZ group. The YZ group saw the emergence of *Carnobacterium* (9.14%) and *Enterobacter* (2.06%), while in the YZD group, *Carnobacterium* (6.98%) decreased but *Citrobacter* (7.60%) appeared. By day 14, in the YS group, *Lactobacillus* (15.12%) and *Carnobacterium* (25.28%) decreased, while *Citrobacter* increased significantly to 41.03%. In the YD group, *Lactobacillus* (36.95%) increased, while *Carnobacterium* (41.95%) and *Desemzia* (9.07%) remained stable, with *Enterobacter* gradually disappearing. In the YZ and YZD groups, *Lactobacillus* remained dominant (72.74, 55.42%), with an opposite trend compared to day 7: the YZ group increased, and the YZD group decreased. In the YZ group, *Carnobacterium* and *Enterobacter* abundances decreased, while *Carnobacterium* in the YZD group increased significantly to 26.02%. At the genus level for fungi (Figure 3J), the distribution of genera with high relative abundances was relatively uniform across all groups and time points, including *Symmetrospora*, *Mycosphaerella*, *Vishniacozyma*, *Alternaria*, *Filobasidium*, and *Sporobolomyces*. Specific fungal genera appeared during aerobic exposure: on day 0, *Botrytis* was enriched in YD (16.46%) and *Knufia* in YZ (9.73%); on day 7, *Acremonium* increased in YZ (11.90%) and *Candida* in YZD (13.37%); by day 14, no specific genera were observed.

### 3.4 Analysis of the correlation between silage quality and microbial communities

**Figure 4:**
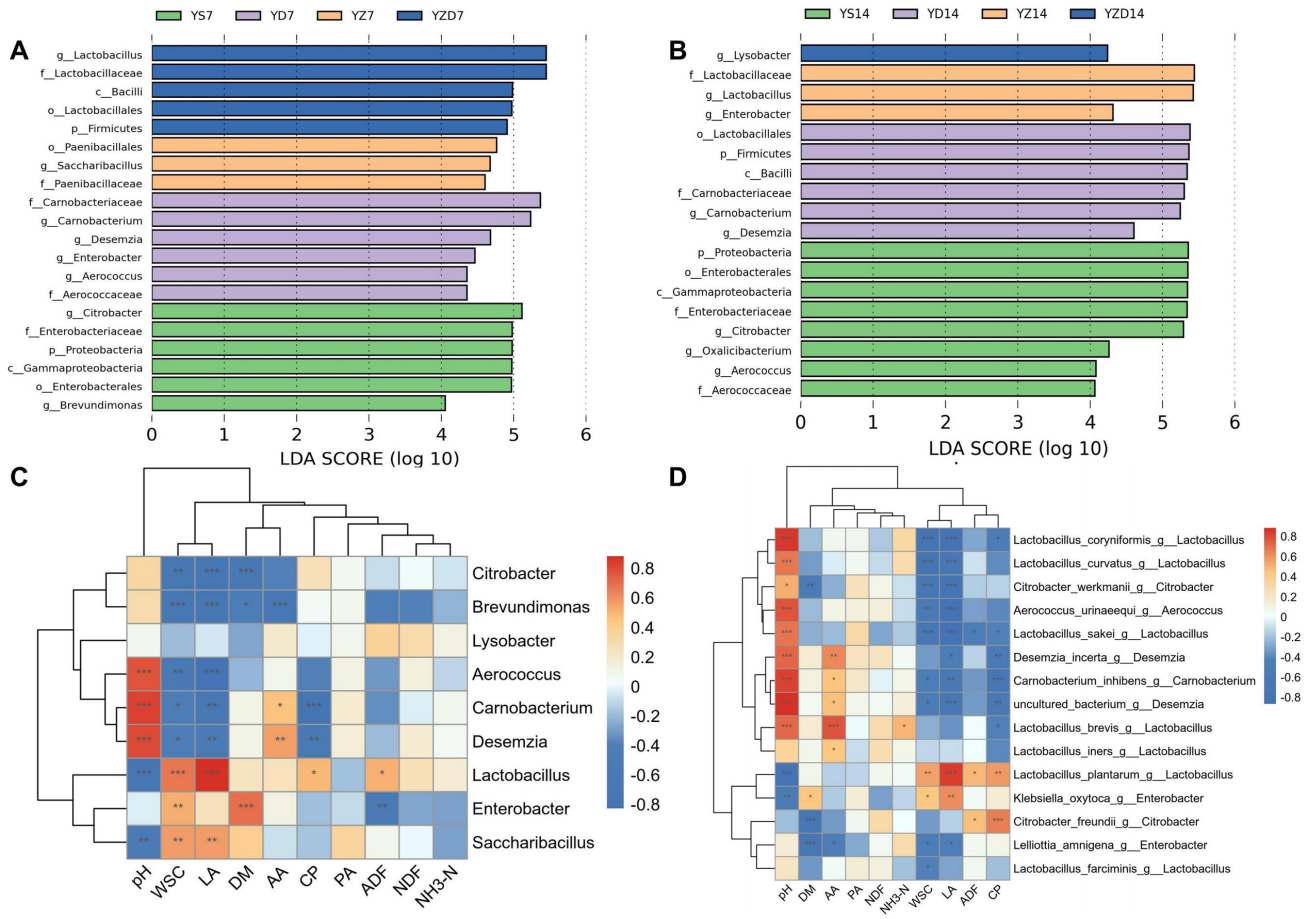
Microbial biomarker screening and correlation analysis during aerobic exposure of silage. LEfSe analysis identifies bacterial genus-level differential biomarkers at 7 **(A)** and 14 days **(B)** of aerobic exposure (LDA score = 4); Spearman correlation heatmap **(C)** shows correlations between bacterial genus-level differential biomarkers and quality traits; the top 30 species-level differential biomarkers, selected from the bacterial genus-level biomarkers, are analyzed in a Spearman correlation heatmap **(D)** for their relationship with quality traits. Statistical significance is indicated as ***p < 0.001, **p < 0.01, *p < 0.05. Red indicates positive correlation, blue indicates negative correlation, and white indicates no correlation. Correlation coefficients (R) are shown as decimals.

LEfSe is typically used to analyze taxonomic groups with significant differences between multi-level species. An LDA score threshold of 4 was set for filtering. The analysis revealed that multiple bacterial species were enriched among the groups during aerobic exposure. At 7 days (Figure 4A), the species significantly enriched in the YS group were *Citrobacter* and *Brevundimonas*, in the YD group were *Carnobacterium*, *Desemzia*, *Enterobacter*, and *Aerococcus*, in the YZ group was *Saccharibacillus*, and in the YZD group was *Lactobacillus*. At 14 days (Figure 4B), the species significantly enriched in the YS group were *Citrobacter*, *Aerococcus*, and *Oxalicibacterium*, in the YD group were *Carnobacterium* and *Desemzia*, in the YZ group were *Lactobacillus* and *Enterobacter*, and in the YZD group was *Lysobacter*.

A correlation heatmap was used to represent the relationship between microbial communities and silage fermentation parameters. LEfSe analysis was combined with Spearman correlation analysis of groups with significant differences at 7 and 14 days, using a correlation coefficient (*R* = ±0.6) and significance level (*p* < 0.001). As shown in Figure 4C, pH was positively correlated with *Carnobacterium*, *Aerococcus*, and *Desemzia*, and negatively correlated with *Lactobacillus*; WSC was positively correlated with *Lactobacillus* and negatively correlated with *Brevundimonas*; LA was positively correlated with *Lactobacillus* and negatively correlated with *Citrobacter* and *Brevundimonas*; DM was positively correlated with *Enterobacter* and negatively correlated with *Citrobacter*; AA was negatively correlated with *Brevundimonas*; CP was negatively correlated with *Carnobacterium*; PA, NH_3_-N, NDF, and ADF had weak correlations with the differential taxa. To further identify key species affecting aerobic exposure, Spearman correlation analysis (*R* = ±0.6, *p* < 0.001) was conducted on the top 30 differential species from the differential taxa. As shown in Figure 4D, pH was positively correlated with *Lv. brevis*, *Loigolactobacillus coryniformis*, *Latilactobacillus sakei*, *Latilactobacillus curvatus*, *Carnobacterium inhibens*, *Desemzia incerta*, and *Aerococcus urinaeequi*, and negatively correlated with *Lpl. plantarum*; DM was negatively correlated with *Citrobacter freundii* and *Lelliottia amnigena*; WSC was negatively correlated with *Lo. coryniformis*, *Lt. curvatus*, *Citrobacter werkmanii*, and *Lt. sakei*; LA was positively correlated with *Lpl. plantarum* and negatively correlated with *Lo. coryniformis*, *Lt. curvatus*, *C. werkmanii*, *A. urinaeequi*, and *Lt. sakei*; CP was positively correlated with *C. freundii* and negatively correlated with *C. inhibens*; Interestingly, AA was positively correlated with *Lv. brevis*.

### 3.5 Metabolite diversity analysis of silage

**Figure 5:**
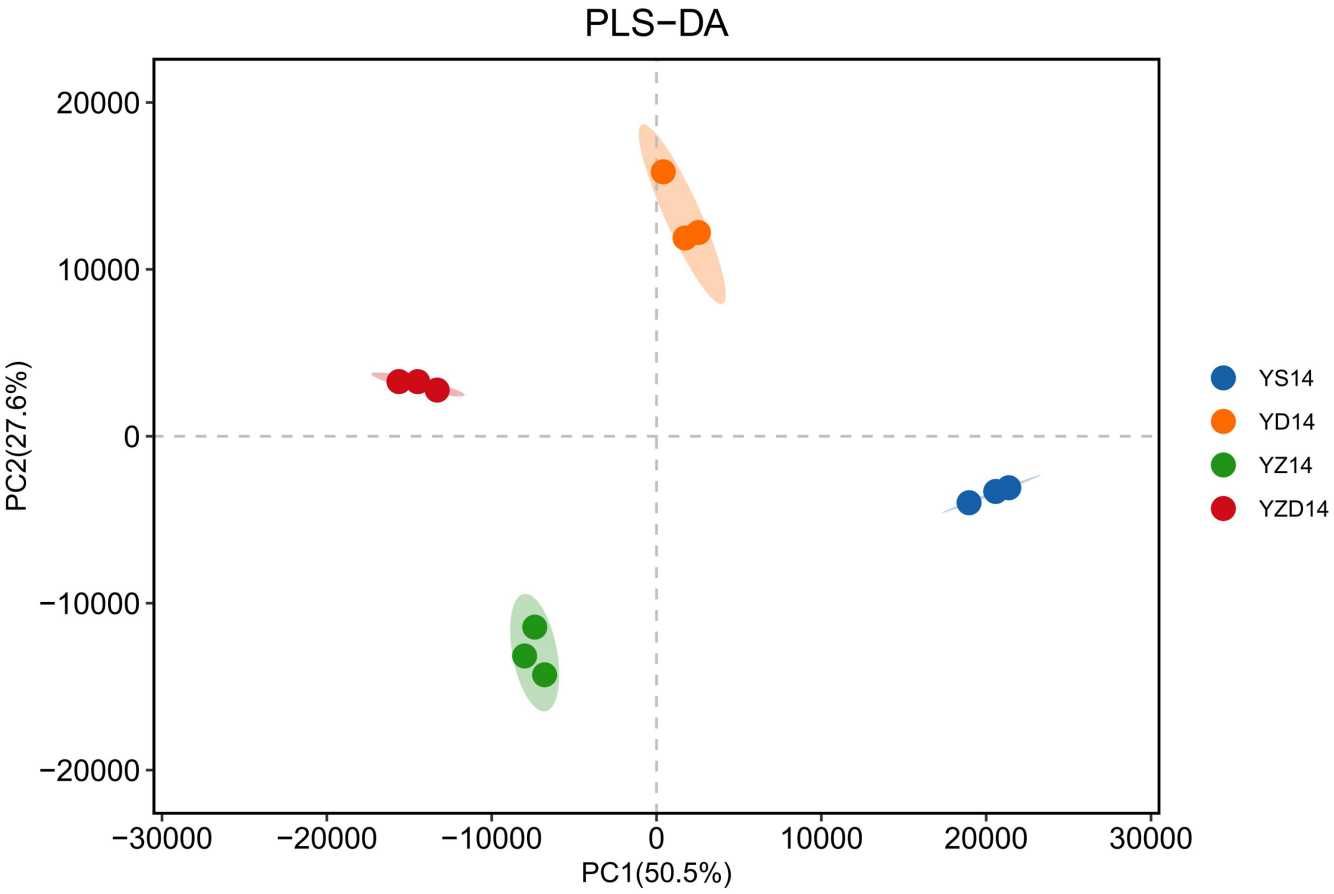
Metabolite diversity analysis during aerobic exposure of silage. The explanation rate (R^2^Y (cum)) and prediction rate (Q^2^ (cum)) are closer to 1, indicating better predictive ability of the model (R^2^Y (cum) = 0.992, Q^2^ (cum) = 0.960).

Non-targeted metabolomics analysis using LC–MS was employed to determine the types and relative concentrations of metabolites in Jerusalem artichoke silage after 14 days of aerobic exposure. A total of 12,338 metabolites were identified. The PLS-DA model effectively demonstrated the separation of metabolites among different groups. A higher R^2^Y (cum) and Q^2^ (cum) value, closer to 1, indicates better explanatory and predictive power of the model. In this study, R^2^Y (cum) = 0.992 and Q^2^ (cum) = 0.96, confirming the reliability of the metabolomics data obtained from the PLS-DA model. As shown in Figure 5, the data within each treatment group exhibited high overlap and good repeatability, with clear separation between groups. Specifically, YD14 is distributed in the first quadrant, YZD14 in the second quadrant, YZ14 in the third quadrant, and YS14 in the fourth quadrant, indicating distinct metabolic profiles among the groups.

### 3.6 Differential metabolite analysis of silage

**Figure 6:**
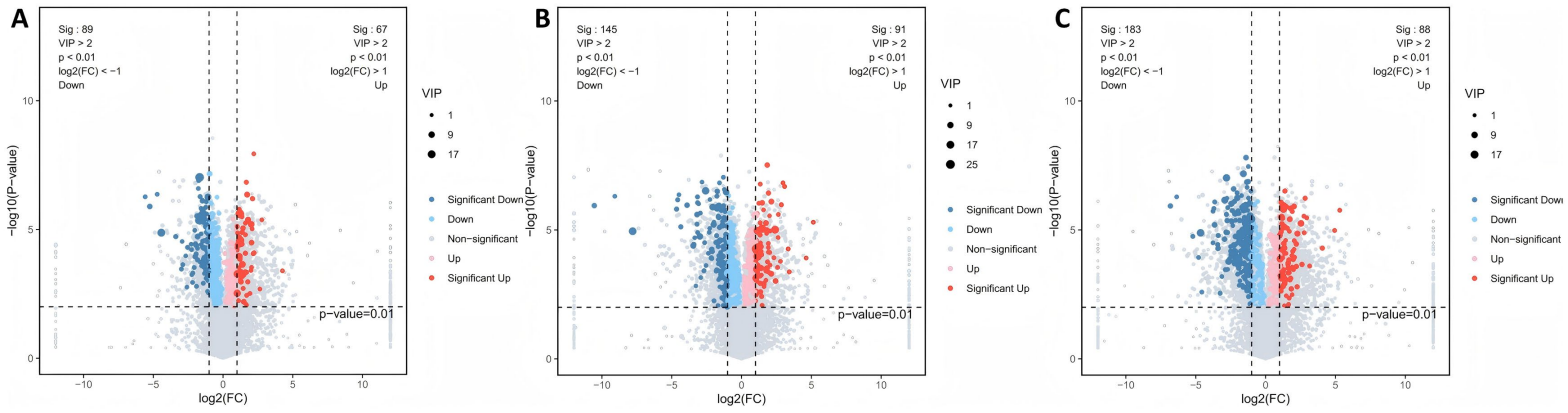
Differential metabolite analysis during aerobic exposure of silage. Volcano plots illustrate changes in differential metabolites between groups: **(A)** YD14 vs. YS14; **(B)** YZ14 vs. YS14; **(C)** YZD14 vs. YS14. Each point represents a metabolite. Red circles indicate significantly upregulated metabolites, blue circles indicate downregulated metabolites, and gray points represent metabolites with no significant difference. Significance criteria: *p* < 0.01, VIP > 1, FC > 2 for upregulation, FC < 0.5 for downregulation.

We used a combination of multivariate and univariate analyses to identify differential metabolites between groups (*p* < 0.01, VIP > 2; FC > 2 indicates upregulation, FC < 0.5 indicates downregulation). Volcano plots illustrate the changes in differential metabolites between groups. Between YD14 and YS14, we identified 156 differential metabolites (67 upregulated, 89 downregulated) (Figure 6A). Upregulated metabolites were primarily flavonoids, while downregulated metabolites were mainly octadecanoids and fatty acids. Between YZ14 and YS14, there were 236 differential metabolites (91 upregulated, 145 downregulated) (Figure 6B). Upregulated metabolites included flavonoids and octadecanoids, whereas downregulated metabolites were primarily fatty acids, eicosanoids, and terpenes. Between YZD14 and YS14, we found 271 differential metabolites (88 upregulated, 183 downregulated) (Figure 6C). Flavonoids were predominantly upregulated, while downregulated metabolites were mainly octadecanoids, fatty acids, and eicosanoids. Compared to the water control, different cold-resistant LAB treatments exhibited varying metabolite profiles, with downregulated metabolites being more prevalent than upregulated ones. This suggests that the water control group experienced more pronounced metabolic shifts during aerobic exposure, indicating a more unstable silage environment.

### 3.7 Analysis of silage metabolic pathways

**Figure 7:**
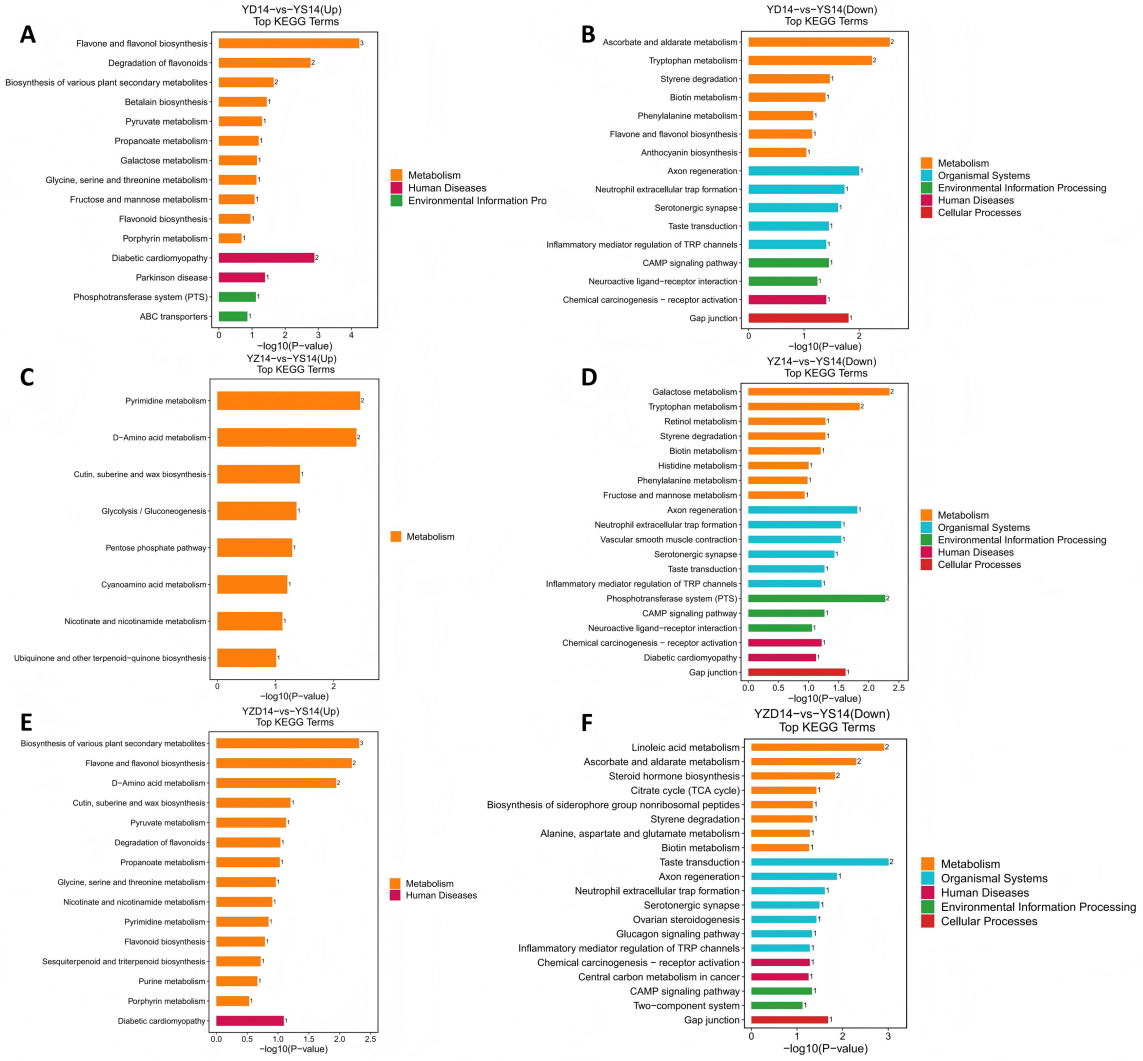
**(A)** indicates the KEGG pathways enriched by DEGs upregulated in YD14 relative to YS14. **(B)** indicates the KEGG pathways enriched by DEGs downregulated in YD14 relative to YS14. **(C)** indicates the KEGG pathways enriched by DEGs upregulated in YZ14 relative to YS14. **(D)** indicates the KEGG pathways enriched by DEGs downregulated in YZ14 relative to YS14. **(E)** indicates the KEGG pathways enriched by DEGs upregulated in YZD14 relative to YS14. **(F)** indicates the KEGG pathways enriched by DEGs downregulated in YZD14 relative to YS14. KEGG metabolic pathway analysis during aerobic exposure of silage. The x-axis represents the −log10 *p*-value of each pathway, while the *y*-axis shows the names of the pathways. The numbers on the bars indicate the number of differential metabolites annotated to each pathway. Bar colors represent different KEGG Level 1 classifications.

The top 20 metabolic pathways with the lowest *p*-values were selected to create enrichment maps for upregulated and downregulated metabolites in the KEGG pathways. Pathways with *p* < 0.01 were considered significantly enriched. For YD14 vs. YS14, upregulated pathways included the biosynthesis of secondary metabolites (such as flavone and flavonol biosynthesis, and flavonoid degradation) and diabetic cardiomyopathy (Figure 7A). Downregulated pathways included carbohydrate metabolism (ascorbate and aldarate metabolism), amino acid metabolism (tryptophan metabolism), and axon regeneration (Figure 7B). For YZ14 vs. YS14, upregulated pathways were nucleotide metabolism (pyrimidine metabolism) and amino acid metabolism (D-amino acid metabolism) (Figure 7C). Downregulated pathways included carbohydrate metabolism (galactose metabolism) and membrane transport (phosphotransferase system) (Figure 7D). For YZD14 vs. YS14, upregulated pathways were the biosynthesis of secondary metabolites (including various plant secondary metabolites, and flavone and flavonol biosynthesis) (Figure 7E), while downregulated pathways included lipid metabolism (linoleic acid metabolism), carbohydrate metabolism (ascorbate and aldarate metabolism), and taste transduction (Figure 7F). The differing pathways enriched with differential metabolites across the groups suggest that various cold-resistant LAB treatments regulate metabolic shifts through different mechanisms.

### 3.8 Correlation analysis between differential metabolites and microbial markers in silage

**Figure 8:**
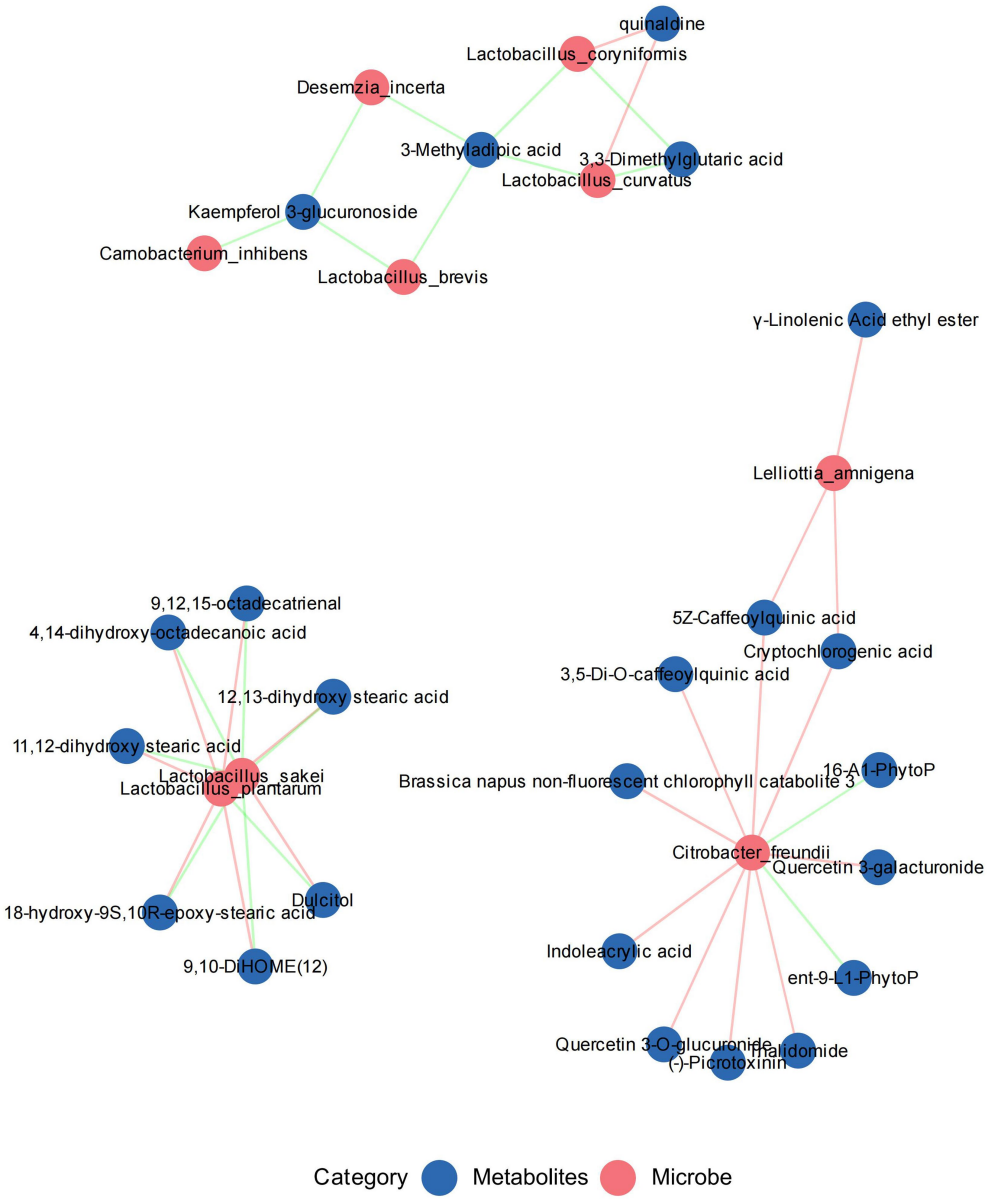
Correlation analysis between differential metabolites and microbial biomarkers in silage feed. Red circles represent microbial biomarkers, blue circles represent differential metabolites. Red lines indicate positive correlations, blue lines indicate negative correlations, and line thickness reflects the magnitude of the correlation coefficient.

We used a correlation network diagram to illustrate the relationships between differential metabolites and bacterial markers after aerobic exposure. For the 14-day aerobic exposure, we selected bacterial markers and the top 10 upregulated and downregulated metabolites from each comparison group, resulting in 36 unique metabolites. Spearman correlation analysis revealed (Figure 8, *R* = ±0.8, *p* < 0.01) that *Lpl. plantarum* and *Lt. sakei* were correlated with seven metabolites. Specifically, *Lpl. plantarum* was positively correlated with 11,12-dihydroxy stearic acid, 12,13-dihydroxy stearic acid, 18-hydroxy-9S,10R-epoxy-stearic acid, 4,14-dihydroxy-octadecanoic acid, 9,10-DiHOME(12), and 9,12,15-octadecatrienal, but negatively correlated with dulcitol. Conversely, *Lt. sakei* exhibited the opposite correlation pattern, indicating antagonistic effects between the two bacteria. *C. freundii* correlated positively with (−)-picrotoxinin, 3,5-di-O-caffeoylquinic acid, 5Z-caffeoylquinic acid, *Brassica napus* non-fluorescent chlorophyll catabolite 3, cryptochlorogenic acid, indoleacrylic acid, quercetin-3-galacturonide, quercetin-3-O-glucuronide, and thalidomide, but negatively with 16-A1-PhytoP and *ent*-9-L1-PhytoP. *L. amnigena* positively correlated with 5Z-Caffeoylquinic acid and cryptochlorogenic acid. *Lo. coryniformis* and *Lt. curvatus* were positively correlated with quinaldine and negatively correlated with 3,3-Dimethylglutaric acid. Kaempferol 3-glucuronoside was negatively correlated with *C. inhibens*, *D. incerta*, and *Lv. brevis*, while 3-Methyladipic acid was negatively correlated with *D. incerta*, *Lv. brevis*, *Lo. coryniformis*, and *Lt. curvatus*.

## 4 Discussion

### 4.1 The inoculation of *Lactiplantibacillus plantarum* slows down aerobic spoilage of feed and preserves its nutritional content

Inoculating with cold-resistant LAB can effectively mitigate the adverse effects of low temperatures on the fermentation process of Jerusalem artichoke silage. Among these, the cold-resistant homofermentative *Lpl. plantarum* GN02 shows enhanced cold fermentation capability (Wei et al., 2024b). During aerobic exposure of the silage, inoculation with cold-resistant LAB helped preserve higher levels of LA, AA and fiber components, maintained higher DM, WSC and CP, and reduced PA, but pH and NH_3_-N still increased as exposure time progressed (Guo et al., 2023; Okoye et al., 2023). When NH_3_-N was expressed on a DM basis, its concentration increased in all treatments during aerobic exposure, and at 7 days the inoculated treatments, especially YZD, showed higher NH_3_-N than the control. However, this increase in NH_3_-N did not correspond to a loss of CP; on the contrary, CP remained comparable to or higher than in the control throughout aerobic exposure, particularly for the *Lpl. plantarum* treatment, which maintained the highest CP at the end of ensiling (0 d of aerobic exposure) (Supplementary Table S1). Importantly, when NH_3_-N was related to total nitrogen, NH_3_-N/TN ranged from 4.53 to 8.97% across treatments and sampling days, which is below the commonly used threshold of 10% TN (≈100 g/kg TN) for well-preserved silage. These values indicate that the observed increases in NH_3_-N represent normal proteolysis within an acceptable range rather than excessive protein degradation that would compromise overall CP content and silage quality (Wilkinson and Davies, 2013; Guan et al., 2020; Hu et al., 2020).

It is generally believed that inoculating with heterofermentative LAB during silage fermentation produces large amounts of AA and PA, which delay nutrient degradation and improve aerobic stability during exposure (Reich and Kung, 2010; Filya and Sucu, 2007). In contrast, inoculation with homofermentative LAB produces high levels of LA and preserved WSC, which can serve as substrates for yeast and other spoilage microorganisms (Andrae et al., 2001; Cole et al., 2007). This can lead to reduced aerobic stability of the silage, increased loss of DM and LA, and significant rises in pH and NH_3_-N (Mugabe et al., 2020), resulting in a serious decline in silage quality. However, this study contrasted with previous research. Inoculation with *Lv. brevis* resulted in significant increases in pH and NH_3_-N during aerobic exposure, failing to maintain a low acidic environment and reducing feed quality. In contrast, the *Lpl. plantarum* inoculated group maintained a favorable acidic environment (pH < 5), preserved higher levels of LA, DM, and WSC. The mixed inoculation group, which ranked just below the *Lpl. plantarum* group, also maintained a low pH and inhibited nutrient degradation. Both the *Lpl. plantarum* and mixed inoculation groups effectively mitigated the adverse effects of cold aerobic exposure on Jerusalem artichoke silage, extending the aerobic stability of the feed. This may be due to the unique winter climate of the Tibetan Plateau, where high altitude and low temperatures affect yeast and spoilage microorganisms, leading to reduced activity and inhibited growth. Consequently, the degradation of silage nutrients through metabolic pathways occurs slowly, resulting in a slower aerobic spoilage process. Similar findings were reported by Zhang (2018) in icegrass silage at an altitude of 3,100 meters on the Tibetan Plateau, where yeast and mold exhibited weak NH_3_-N production, and spoilage occurred slowly during aerobic exposure. Therefore, aerobic spoilage of silage on the Tibetan Plateau may be directly related to altitude and temperature. The slow aerobic spoilage process might be common in high-altitude areas.

### 4.2 Inoculating with *Lactiplantibacillus plantarum* helps maintain microbial diversity and beneficial bacteria abundance in the feed while inhibiting the growth of harmful bacteria

The growth and proliferation of harmful bacteria were the primary factors leading to aerobic spoilage. Microbial diversity analysis indicated that the diversity index of the CK remained high during aerobic exposure, suggesting that inoculating with psychrotolerant LAB can inhibit the proliferation of spoilage bacteria, consistent with the findings of Wang et al. (2021). The group inoculated with *Lpl. plantarum* showed a lower bacterial diversity index, indicating that low temperatures and inoculation with *Lpl. plantarum* effectively suppressed the growth of bacterial species. The slight increase in richness index during the later stages of aerobic exposure in the mixed group may be due to the proliferation of a small number of psychrotolerant aerobic bacteria, which contrasts with previous studies (Guan et al., 2021; Yin et al., 2023). This phenomenon may be attributed to the ability of low temperatures to inhibit the reproduction of most aerobic bacteria, resulting in slower consumption of LA, a substrate for aerobic bacteria (Keshri et al., 2018), and a gradual rise in pH, further suppressing the activity of pH-sensitive aerobic bacteria. Temperature and pH are likely critical factors influencing bacterial proliferation during low-temperature aerobic exposure (Shan et al., 2021). There was little difference in fungal community richness across groups during the aerobic exposure phase, with fungal diversity stabilizing in the later stages, indicating minimal impact from inoculation on fungal communities. The specific reasons for this are unclear. Compared to bacteria, fungi are more strongly inhibited by low temperatures, aligning with the findings of Lei et al. (2022). Changes in microbial communities explain the varying degrees of silage susceptibility to aerobic conditions. Principal Coordinate Analysis (PCoA) of bacterial communities revealed that the type of inoculant and duration of aerobic exposure influenced microbial community changes during the aerobic exposure of Jerusalem artichoke silage. The two groups inoculated with *Lpl. plantarum* showed more similar bacterial community structures to each other, while being more distinct from the CK and the group inoculated with *Lv. brevis*. This suggests that bacterial community changes were more consistent in the groups inoculated with *Lpl. plantarum*, which may be due to the effects of low temperature and pH. In contrast, fungal PCoA analysis showed disorder, with bacterial communities being more affected by inoculation and exposure time than fungal communities.

Changes in microbial community abundance indicate that Firmicutes consistently dominated in the cold-resistant LAB inoculated group compared to the control, consistent with the results of Auerbach and Theobald (2020a). In the *Lpl. plantarum* inoculated and mixed groups, *Lactobacillus* abundance remained high. This may be due to the isolation and anaerobic nature of some LAB strains (Jiang et al., 2020), which delayed the decline in *Lactobacillus* levels, or it could be that low temperatures restricted bacterial proliferation, leading to slower community changes. Similar to the end of the fermentation phase, *Carnobacterium* and *Desemzia* continued to dominate in the non-inoculated and *Lv. brevis* inoculated groups. Their abundance increased slowly with extended exposure time, possibly due to the onset of spoilage during fermentation, which led to the proliferation of spoilage bacteria. Although AA and PA also accumulated, their levels did not reach the threshold needed to inhibit spoilage bacteria, and environmental factors further promoted their growth. As at the end of fermentation, Ascomycota and Basidiomycota remained the dominant fungal phyla in all groups, with *Symmetrospora*, *Mycosphaerella*, *Vishniacozyma*, and *Alternaria* as the predominant genera. There were minimal changes in fungal phyla and genera, indicating that fungal activity was suppressed and that fungi could not carry out extensive life processes under low-temperature aerobic conditions. Previous studies have shown that silage inoculated with *Lpl. plantarum* has poorer aerobic stability during exposure, with higher abundances of Ascomycota and Basidiomycota compared to other groups (Auerbach and Nadeau, 2020b). The abundance of yeasts is also higher, with lactate-utilizing yeasts contributing to reduced aerobic stability (Liu et al., 2018a). In this study, fungal community abundance did not exhibit significant fluctuations, indicating that low-temperature environments effectively suppress aerobic spoilage of silage. Given the unique environmental conditions, further research is needed across various altitudes, temperatures, and crop types.

The correlation between microbial markers and quality indicators provides a clear view of their interactions. The correlation heatmap shows that *Lactobacillus* helps preserve LA and WSC while maintaining a low pH in the feed. In contrast, *Aerococcus*, *Carnobacterium*, and *Desemzia* are associated with increased pH, and *Citrobacter* is linked to decreased DM. These microbes disrupt the feed’s microbial balance and create environments conducive to their own growth (Wang Z. et al., 2022). While genus-level correlation analysis has limitations, species-level analysis can further clarify relationships with quality indicators. *Lactobacillus* species, particularly *Lpl. plantarum*, were crucial for maintaining an acidic environment and preserving LA and WSC during aerobic exposure. Although *Lv. brevis* preserves more AA, it also causes a rise in pH. Similar to the fermentation phase, spoilage microbes such as *Lo. coryniformis*, *Lt. curvatus*, *C. werkmanii*, *A. urinaeequi*, *Lt. sakei*, *D. incerta*, and *C. inhibens* produce volatile organic acids or CO_2_ from LA and WSC in aerobic conditions, while *C. freundii* and *L. amnigena* degrade DM, depleting the feed’s nutrients and increasing pH, which shortens the aerobic exposure time. Thus, inoculating with *Lpl. plantarum* GN02 during the aerobic exposure phase of Jerusalem artichoke silage can ensure a longer aerobic exposure time, inhibit the breakdown of LA, WSC, and DM by aerobic microbes, preserve more nutrients, and maintain an acidic environment.

### 4.3 The inoculation of *Lactiplantibacillus plantarum* secretes antioxidant substances that establish a defense mechanism against spoilage microbes

In recent years, metabolomics has emerged as a powerful tool for elucidating the complex metabolic processes in silage, especially during the aerobic exposure phase. This approach provides valuable insights into the metabolic adaptations occurring within silage (Liu et al., 2018b; Guan et al., 2022). Metabolite analysis of Jerusalem artichoke silage during winter aerobic exposure revealed differences in metabolite composition between LAB-inoculated and non-inoculated groups. Flavonoids were significantly upregulated in the inoculated group, potentially indicating aerobic spoilage. This upregulation may suggest that LAB secretes flavonoids to counteract environmental stress and spoilage microbes, providing some inhibition of aerobic spoilage (Corsetti et al., 2004; Wang W. et al., 2022). Fatty acids were among the major downregulated metabolites across all groups, possibly due to changes in microbial activity during aerobic exposure. LAB may be adjusting the internal antioxidant response system of the silage environment, enhancing the production of other antioxidants or activating antioxidant enzymes (Rodríguez et al., 2009). The upregulation of flavonoids supports this view, indicating substrate consumption, while LAB may use stored fatty acids as energy and raw material (Guan et al., 2020). In the *Lpl. plantarum* inoculated group, eicosanoids and terpenes were also downregulated. This may be due to certain microbes preferring these compounds as substrates under aerobic conditions, leading to different metabolic pathways and products compared to anaerobic fermentation, affecting the production of eicosanoids and terpenes (Zhang et al., 2020; Sehl et al., 2005). Notably, octadecanoids were upregulated in the *Lpl. plantarum* inoculated group but downregulated in the other two groups. Previous research suggested that the *Lv. brevis* inoculated group experienced fermentation failure, leading to spoilage bacteria proliferation and silage deterioration (Wei et al., 2024b). The production of octadecanoids may indicate the onset of silage spoilage, accelerating the process as these compounds are consumed, similar to their antioxidant role in plants, where they protect cells and mitigate damage from aerobic spoilage bacteria. They may also regulate LAB’s response to stress, enhancing resistance (Tayeh et al., 2016; Miersch and Wasternack, 2000).

### 4.4 Inoculation with *Lactiplantibacillus plantarum* inhibits pathways involved in nutrient degradation

During the silage process, microorganisms may modify their secondary metabolism pathways to adapt to new environmental conditions and compete more effectively, producing secondary metabolites with antibacterial and antioxidant activities (Corsetti et al., 2004). In this study, differential metabolites in the *Lv. brevis* and mixed groups were enriched in the pathway for the biosynthesis of other secondary metabolites, which was upregulated. This indicates that under aerobic exposure, microorganisms experienced oxidative stress, altering their metabolic pathways. Unlike other groups, the *Lpl. plantarum* group showed upregulation in nucleotide metabolism and amino acid metabolism pathways, which are involved in protein synthesis (McEniry et al., 2008). The upregulation of these pathways may suggest that fewer spoilage microorganisms accumulated post-fermentation in the *Lpl. plantarum* group, preserving more amino acids and proteins despite the aerobic environment. Carbohydrate metabolism is a crucial pathway in silage. During aerobic exposure, spoilage microorganisms oxidize pyruvate or lactic acid to CO_2_ and H_2_O, reducing the concentration of carbohydrates and other nutrients in the feed (Keshri et al., 2018). In this study, carbohydrate metabolism was downregulated in all treatment groups, indicating that inoculation with cold-resistant LAB effectively inhibits the degradation of carbohydrates during aerobic exposure, preserving nutrients.

### 4.5 Correlation analysis suggests that octadecanoid compounds may play a role in the aerobic defense mechanisms of *Lactiplantibacillus plantarum*

Correlation analysis between microorganisms and metabolites is a crucial method for understanding the ecological roles of microorganisms in the silage process and how they impact silage quality and stability. *Lpl. plantarum* showed a positive correlation with six octadecanoid metabolites, which were identified as upregulated in the differential metabolite analysis. Studies suggest that octadecanoids are a broad category of compounds associated with oxidative stress (Ahanchi et al., 2018; Munné-Bosch and Alegre, 2002). Their increased levels may indicate heightened oxidative stress during silage, and their potential antioxidant properties suggest a microbial response to oxidative stress or a defense mechanism. The positive association with *Lpl. plantarum* suggests direct or indirect production and a role in modulating its stress responses. Octadecanoids are relatively under-researched in silage, and their specific roles require further detailed study. *Lt. sakei* showed a negative correlation with these octadecanoids, indicating potential antioxidant activity and inhibition of *Lt. sakei* growth. *C. freundii* was positively correlated with nine metabolites, including indoles, flavonoids, and polyphenols, known for their defensive roles against oxidative stress and microbial invasion (Agati et al., 2012; Pandey and Rizvi, 2009; Khan et al., 2013). These metabolites’ upregulation suggests a defensive response to *C. freundii*, a significant aerobic spoilage bacterium in the low-temperature silage exposure of Jerusalem artichoke. *Lv. brevis* showed no significant correlation with many metabolites, indicating that its activity might be suppressed under aerobic conditions, low temperatures, pH variations, and competition with other microbial populations, aligning with observations of its declining abundance during aerobic exposure.

## 5 Conclusion

This study demonstrated that cold-resistant *Lactiplantibacillus plantarum* GN02 effectively improved the aerobic stability of Jerusalem artichoke silage under low-temperature conditions on the Qinghai-Tibet Plateau. The inoculant maintained lower pH, higher lactic acid, dry matter, and water-soluble carbohydrate levels, while promoting antioxidant metabolite accumulation and suppressing spoilage bacteria. Upregulation of amino acid and nucleotide metabolism, along with downregulation of carbohydrate degradation, contributed to nutrient preservation. In contrast, *Lv. brevis* accelerated spoilage, and the mixed inoculation showed moderate effects. Overall, inoculation with *Lpl. plantarum* GN02 significantly delayed aerobic deterioration and extended the storage life of silage in plateau environments.

## Data Availability

The datasets presented in this study can be found in online repositories. The names of the repository/repositories and accession number(s) can be found at: https://www.ncbi.nlm.nih.gov/, PRJNA1088907 https://www.ncbi.nlm.nih.gov/, PRJNA10889919.

## Footnotes

1
http://cloud.oebiotech.com
